# A journey towards whole water certified reference materials for organic substances: measuring polycyclic aromatic hydrocarbons as required by the European Union Water Framework Directive

**DOI:** 10.1007/s00216-021-03200-2

**Published:** 2021-02-18

**Authors:** Ioannis Dosis, Marina Ricci, Håkan Emteborg, Hendrik Emons

**Affiliations:** 1 European Commission, Joint Research Centre (JRC), 2440 Geel, Belgium; 2 German Environment Agency, Wörlitzer Platz 1, 06844 Dessau-Roßlau, Germany

**Keywords:** Environmental Quality Standards (EQS), Humic acids, PAHs, Priority substances, Environmental monitoring, Surface water analysis

## Abstract

In 2000, the Water Framework Directive (WFD) came into force in the European Union with the aim of protecting and improving water quality. The priority substances established to be monitored are predominantly organic compounds, for which the WFD sets the requirement of ‘whole water sample’ analysis. This legislative requirement poses analytical challenges for the monitoring laboratories as well as technical challenges for reference materials producers. In the past, there were attempts to produce reference materials as quality assurance/quality control tools for measuring organic priority substances in whole water. A critical reflection on the approaches and solutions applied to prepare such kind of matrix reference materials is presented along with a discussion on the difficulties encountered by the analytical laboratories in analysing such complex matrices. The Certified Reference Material (CRM) ERM-CA100 can be considered as a pioneer for a ‘whole water’ CRM (containing humic acids) and has been designed for the analysis of polycyclic aromatic hydrocarbons (PAHs). Further developments seem to be necessary to upgrade the design towards a CRM which will also include suspended particulate matter, another basic constituent of natural surface water samples.

**Supplementary Information:**

The online version contains supplementary material available at 10.1007/s00216-021-03200-2.

**Supplementary Information:**

The online version contains supplementary material available at 10.1007/s00216-021-03200-2.

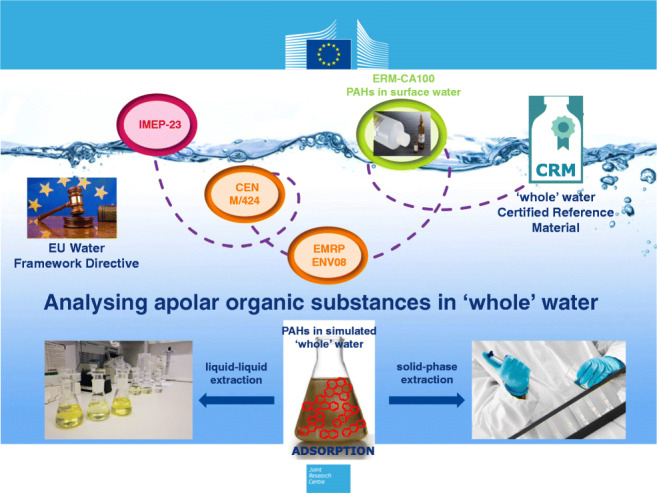

## Introduction

Polycyclic aromatic hydrocarbons (PAHs) exhibit a high affinity to natural organic and suspended particulate matter in aqueous environments [1]. Natural organic matter is mainly derived from plant decay and is present in most water bodies as soluble and colloidal humic (including fulvic) substances or acids (also referred to as dissolved organic matter consisting of mixtures of macromolecules smaller than 0.45 μm). The widespread presence of PAHs combined with their lipophilicity and bioaccumulation potential contribute to their relevance as environmental pollutants. Health effects vary, also related to molecular weight: lower mass PAHs may exhibit acute toxicity for aquatic organisms, while some of the higher mass PAHs are classified as known, possible, or probable carcinogenic to humans (group 1, 2A or 2B) by the International Agency for Research on Cancer [2, 3].

The protection of environmental water bodies in the EU falls under the scope of the Water Framework Directive (WFD) [4], which came into force as a pollution control strategy in 2000. It requires EU Member States to assess, monitor and control a number of Priority Substances (PS) in all inland and coastal waters. Environmental Quality Standards (EQS) were established for all PS as thresholds which must not be exceeded to protect human life and the environment [5, 6]. The large majority of the PS are apolar organic substances, among them eight PAHs: naphthalene, anthracene, fluoranthene, benzo[*b*]fluoranthene, benzo[*k*]fluoranthene, benzo[*a*]pyrene, indeno[1,2,3-*cd*]pyrene and benzo[*ghi*]perylene. The Directive 2013/39/EU [5] regulates that benzo[*b*]fluoranthene, benzo[*k*]fluoranthene, indeno[1,2,3-*cd*]pyrene and benzo[*ghi*]perylene, while still included in the PS list, are not subjected to mandatory monitoring anymore and benzo[*a*]pyrene is henceforth to be considered as a marker for all of them. The same Directive additionally assigns biota EQS to some PS, including fluoranthene and benzo[*a*]pyrene. Another Directive from 2009 has set challenging minimum performance criteria for the methods of analysis to be applied in the water status monitoring programmes of the PS (e.g. limit of quantification of the analytical methods applied must be 30% of the EQS). The aim is to secure that Member States report valid and comparable results, through the participation of their appointed expert laboratories in proficiency tests as well as through the use of reference materials (RM) ‘representative of collected samples which contain appropriate levels of concentrations in relation to environmental quality standards’ [7].

All eight PAHs regulated by the WFD were included in the design and production of ERM-CA100 (PAHs in surface water), which was carried out by the Joint Research Centre (JRC) of the European Commission. ERM-CA100 is the first CRM for an organic PS in water available on the market to date.

A specific challenge for the CRM project was the fact that the WFD established the requirement of ‘whole water sample’ analysis for all organic PS. This is adding significant complexity to the already non-trivial design and development of CRMs for lipophilic organic substances in water [8–13]. The ‘whole water’ designation is aiming at the inclusion of suspended particulate matter (SPM) and/or dissolved organic matter to properly account for the pollutant’s portion adsorbed on them (especially for apolar compounds) and is posing a real analytical challenge for the monitoring laboratories [14–18]. Despite some papers reporting the analysis of unfiltered water samples [19, 20], it is recurrent to encounter scientific literature reports presenting monitoring results for organic PS obtained with analytical methods which foresee a water filtration step before the final detection [21–23]. This implies that the methods used are not suitable for monitoring under the WFD. The obtained results should be considered only as fractional (i.e. providing part of the load of contaminants, namely the one in the dissolved phase) and non-compliant with the WFD criteria. It has to be reminded that in 2015 CEN/TC230 (European Committee for Standardization, Technical Committee Water Analysis) released four documentary standards, developed under mandate M/424 of the European Commission, for the analysis of PAHs, tributyltin (TBT), organochlorine pesticides (OCP) and polybrominated diphenyl ethers (PBDEs) applicable to water samples containing up to 500 mg/L of SPM [24–27].

The preparation of water reference materials (RMs) for selected organic substances (including PAHs) was also one of the objectives of the project ‘Traceable measurements for monitoring critical pollutants under the European Water Framework Directive (ENV08)’ of the European Metrology Research Programme (EMRP) which finished in 2014 [13, 28].

The aims of this paper are to discuss the analytical and technical challenges when approaching the regulatory relevant ‘whole water’ analysis of organic priority substances and to share some insights, outcomes and findings acquired during the certification of ERM-CA100.

## Paving the way towards a whole water CRM

The absence of a ‘whole’ water CRM for organic PS, as the WFD requires, is a problem to which a complete and final solution has yet to be presented. Reference material producers and proficiency testing (PT) providers have been attempting to resolve the matter for many years; however, they offered only ‘partial’ solutions until now. The main challenges and obstacles preventing a satisfactory realisation of a homogeneous and stable (whole) water CRM for organic compounds are known and have been reviewed already in several publications [8–12].

A clear definition of what constitutes a ‘whole’ water sample is not provided in the WFD. In one of the Guidance documents developed for the implementation of the WFD (Guidance 19 on surface water chemical monitoring under the WFD [29]), it is reported that ‘whole’ water is a ‘synonym for the original water sample and shall mean the water sample when solid matter and the liquid phase have not been separated’.

The PT samples prepared in 2008 for the interlaboratory comparison (ILC) IMEP-23—the eight EU-WFD priority PAHs in water in the presence of humic acid—could be considered, according to the authors’ knowledge, as the first attempt to provide a whole water RM for organics (Table 1) [30]. The International Measurement Evaluation Programme (IMEP) [34] was a programme run by the JRC of the European Commission. Those ILCs were organised using reference values with associated uncertainties for the evaluation of participants’ results. The IMEP-23 PT samples consisted of two water samples, each of 500 mL, to be reconstituted at the laboratory’s premises via the spiking with 1 mL of a humic acid solution and 1 mL of a PAH solution. Even though the assignment of reference values was carried out for these samples, they could not be considered as CRMs, because stability was assessed solely for the duration of the ILC, i.e. 2 months, and no certificate was issued. Nevertheless, the IMEP-23 samples could be considered as the ‘prototype’ of CRM ERM-CA100.

**Table 1.** Whole water RMs for organic analytes

	Type of RM	Format	Water matrix	Analytes / concentration range	Other matrix components / concentration	Stability study duration (months)	Reference
IMEP-23	PT sample	To be reconstituted	Filtered groundwater	8 PAHs/69-126 ng/L	HA/5 mg/L*	2	[30]
EMRP ENV08	PT sample	Ready-to-use	Mineral water	8 PAHs/20.6-367 ng/L	Sample 1: HA/5 mg/L* + SPM / 38.6 mg/L	1.5	[13,28,32]
				7 PAHs/5.7-94.5 ng/L	Sample 2: SPM/20.2 mg/L		
				6 PBDEs/0.032-5.90 ng/L	Sample 1: HA/5 mg/L* + SPM/193.5 mg/L		
				0.004-0.77 ng/L	Sample 2: SPM/25.2 mg/L		
				TBT / 4.07 ng/L	Sample 1: HA/5 mg/L* + SPM 8.2/mg/L Sample 2: SPM/21 mg/L		
CEN TC230 M424	Collaborative trial sample	Ready-to-use	Mineral water	12 PAHs/3.64-94.3 ng/L	Sample 1**: SPM/20 mg/L	1.5	[24–27,33]
				8 PAHs/27.2-476.3 ng/L	Sample 2**: SPM/200 mg/L		
			Mineral water	TBT/3.8 ng/L	Sample 1**: SPM/20 mg/L		
				3.4 ng/L	Sample 2**: SPM/200 mg/L		
			Surface water	Organochlorine Pesticides/0.0139-0.4059 μg/L	Sample 2**: SPM/20 mg/L		
				0.0621-0.5065 μg/L	Sample 3**: SPM/200 mg/L		
			Mineral water	PBDEs/0.032-5.82 ng/L	Sample 1**: SPM/20 mg/L		
				0.072-0.761 ng/L	Sample 2**: SPM/200 mg/L		
ERM-CA100	Certified Reference Material	To be reconstituted	Surface filtered water	8 PAHs/29-104 ng/L (except Naphtalene 1.21 μg/L)	HA/20 mg/L*	18 (stability confirmed since 2016)	[36]

*PT* proficiency testing, *PAHs* Polycyclic aromatic hydrocarbons, *PBDEs* Polybrominated diphenylethers, *TBT* Tributyltin, *HA* Humic acids, *SPM* Suspended particulate matter

*The concentration of HA is expressed as mg carbon per liter

**As defined in the CEN standards

In parallel to the production of ERM-CA100, other RMs targeting a whole water matrix were prepared within the EMRP ENV08 project for PBDEs, PAHs and TBT [13, 28, 31]. However, none of these RMs could be classified as certified, given that no rigorous value assignment was carried out (in this case stability was assessed for a period of 7 weeks). Looking further into differences between the materials, it should be pointed out that the water matrix of the EMRP ENV08 RMs was mineral water (commercially available), while the matrix of ERM-CA100 is surface water, thus resembling more closely the water types targeted by the WFD. In addition, the ENV08 materials were off-the-shelf ready-to-use items, while ERM-CA100 has to be reconstituted at the laboratory’s premises. The two approaches present both advantages and disadvantages. While an off-the-shelf material could be handled more easily, the kit option may introduce more flexibility regarding sample size and number of replicates. Two types of test RMs were developed within the ENV08 project: one containing only SPM at a concentration between 20 and 25 mg/L (depending on the test material) and a second containing both SPM (at two levels of concentrations up to 200 mg/L depending again on the test material) and HA at a 5 mg/L level (Table 1). Those RMs were used in an interlaboratory comparison (after assessing homogeneity and stability for the purpose of the study), further proving the validity of the approach used in their preparation [31]. The organic contaminants were introduced in these water RMs via the SPM itself. For that, already existing soil and sediment RMs (further milled) were used, in which the substances of interest were present [13].

‘Adapted’ ENV08 test materials containing only SPM at 20 and 200 mg/L level, hence excluding the HA, were used in the interlaboratory trials for the validation of the methods in the ‘water quality’ standards published in 2015 by CEN/TC230 [24–27]. These methods, based on the use of solid-phase extraction (SPE) disks, are applicable for water samples with a SPM content up to 500 mg/L [32]. The test materials for the trials on PAHs, TBT and PBDEs were prepared by the JRC, while the test material for OCP was prepared by the IWW Water Centre (Germany), based on the experience gained during the EMRP ENV08 project. Only suspended particulate matter was considered in these standards as a constituent of the surface waters, besides the liquid phase (Table 1). Natural colloids (such as humic acids) were not taken into account, although they may significantly contribute to the adsorption of organic substances [35] and to the complexity of the sample extraction and pre-treatment, depending on their load [36].

## Requirements and challenges in the preparation of whole water CRMs for apolar organic substances

**Fig. 1:**
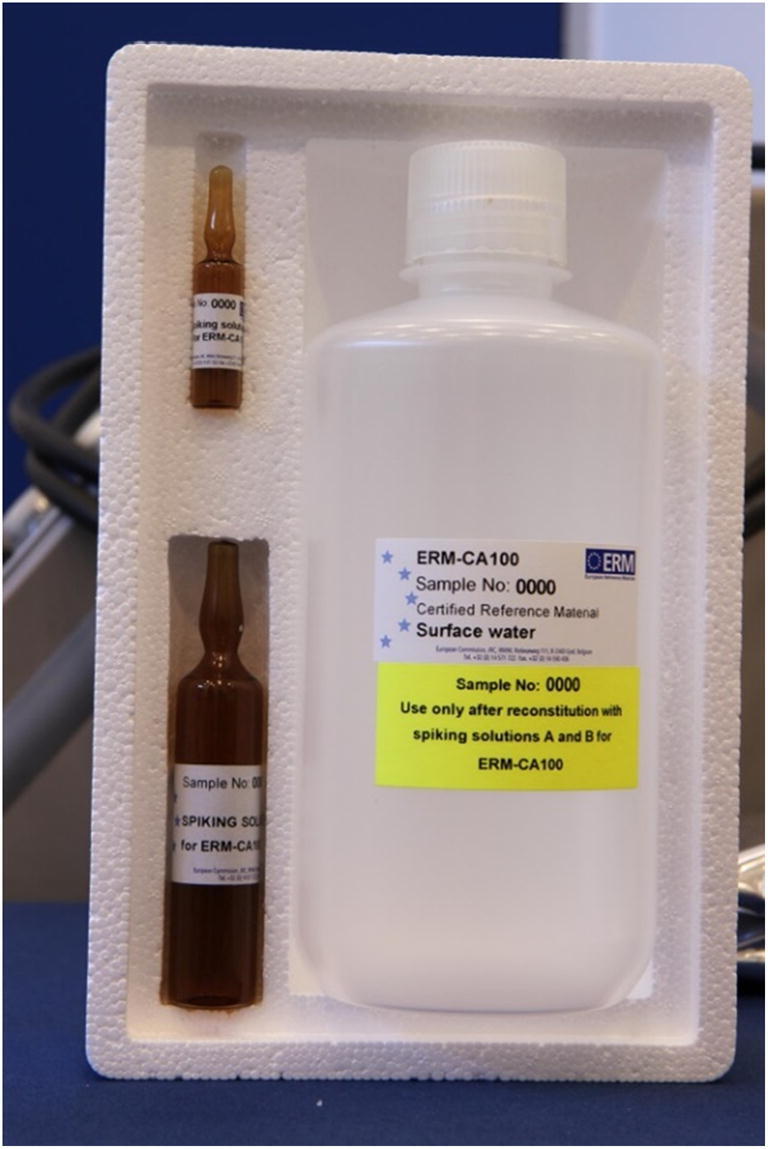
One unit of ERM-CA100

The ERM-CA100 project (PAHs in surface water) started in 2010 and the CRM was released by the JRC in 2015 as a ‘kit’ [33]. This CRM contains three items: a bottle of at least 1 L of surface water, an amber glass ampoule ‘Spiking solution A’ containing at least 24 mL of HA solution in water and an amber glass ampoule ‘Spiking solution B’ containing at least 2.3 mL of PAHs solution in acetonitrile (see Fig. 1).

This CRM ‘kit’ allows the preparation of two independent water samples of 500 mL each, with a concentration range of the target WFD PAHs between ca. 30 and 100 ng/L (except naphthalene, certified at 1.21 μg/L). An approximate concentration of carbon equal to 0.02 g/L is achieved in the samples by the addition of the HA solution. The humic acids act as simulant of dissolved organic matter mimicking a whole water sample. The concentration of 20 mg carbon/L is considered to be on the high side of the spectrum for a natural organic matter presence in surface waters [37]. The CRM, when reconstituted, shows indeed a brownish colour indicating a high load of organic matter.

The certification of ERM-CA100 was conducted under accreditation to ISO Guide 34 [38]. Homogeneity and stability [39] were assessed for all PAHs in the final reconstituted reference material. The set of certified values is shown in Table 2. A full report containing all details of the production of ERM-CA100 [33] is available via the CRM online catalogue at https://crm.jrc.ec.europa.eu/.

**Table 2:** Certified values with uncertainties for ERM-CA100

PAH	Number of accepted datasets	Certified value	*U*_CRM_^1)^
Naphthalene	8	1.21 μg/L	0.13 μg/L
Anthracene	8	91 ng/L	11 ng/L
Fluoranthene	10	104 ng/L	11 ng/L
Benzo[*b*]fluoranthene	7	32 ng/L	9 ng/L
Benzo[*k*]fluoranthene	8	38 ng/L	9 ng/L
Benzo[*a*]pyrene	7	42 ng/L	8 ng/L
Indeno[1,2,3-*cd*]pyrene	6	29 ng/L	7 ng/L

^1)^Expanded (*k* = 2) uncertainty

An additional information value of 31 ng/L (without uncertainty) is assigned to benzo[*ghi*]perylene

The production of ERM-CA100 did not exhibit particular problems at the processing step, perhaps with the exception of the humic acid preparation. As already noted, the load of organic carbon in the matrix is quite high and the dissolution of the humic acid (purchased by Sigma-Aldrich N.V as technical grade with 20% of residue impurities) to obtain a homogeneous solution required some technical work-around and a lot of patience.

The introduction of a HA constituent (without including SPM) had been designed as a step towards a whole water sample, thus already presenting adsorption issues for hydrophobic compounds. It may not impact the sample pre-treatment as much as in the case of a matrix with SPM. On the other hand, one could argue that SPM could easily be filtered off and analysed separately from the water phase (afterwards providing an aggregated result for the total load of the contaminants), while dissolved organic matter such as humic acid cannot be separated by filtration and has to be handled together with the water phase.

Long-term stability is a necessary prerequisite for a CRM and in the case of the ERM-CA100 it was assessed during a period of 18 months. In contrast to the EMRP ENV08 PT samples described in the previous chapter, a reconstitution step was deemed necessary to avoid the risk of compromising the homogeneity and stability of the material. Apolar organic substances like PAHs tend to adsorb onto the walls of the containers (and onto the humic acids) rather than remaining in the water phase [31, 40]. Obviously, this could negatively influence the homogeneity as well as the stability (especially the long-term stability) of the CRM. Indeed, adsorption of the PAHs onto the humic acid puts into motion a complex system for which the equilibrium can change over time [1, 9–12, 41].

The reconstitution protocol to be followed was developed on the basis of a thorough study on the adsorption behaviour of PAHs onto humic acids, carried out during a PhD thesis project [15] and further refined to enable the characterisation study. No complaints were received during the characterisation exercise by any of the participating laboratories, confirming its simple application and providing reassurance with respect to the future use of the CRM by laboratories.

The fact that the CRM has to be ‘created’ at the laboratory’s premises, thus presumably introducing some additional unit variation, has been duly taken into consideration at the stage of the homogeneity assessment, which was run on reconstituted samples. Uncertainty contributions related to homogeneity range between 1.7 and 4.7% (Supplementary Information (ESM), Table S1). Considering that PAH analysis has reached very high accuracy levels, these variations may seem excessive for a CRM but it can be rationalised by the additional uncertainty contribution due to the reconstitution. However, they were deemed acceptable for such a multi-phase matrix design.

The assessment of the stability of ERM-CA100 was based on the stability of the CRM components (water, humic acid solution and PAHs solution), i.e. the temperature shifting of the isochronous studies was performed with the separate components [39], while the measurements were conducted on the reconstituted material. Especially in the case of the HA solution, long-term storage might induce changes (e.g. conformational) which could influence the adsorption properties towards the PAHs when the CRM unit is reconstituted. It was therefore important to investigate a possible stability effect and include it in the uncertainty of the certified values.

The uncertainty contributions of stability, being higher for the PAHs present at lower concentrations (30–40 ng/L, see ESM Table S1), include the variation of the reconstitution steps as well (thus doubling an uncertainty contribution already present in the estimation of the uncertainty related to the CRM homogeneity).

The preparation of a ‘whole water’ CRM for the monitoring of organic PS according to the WFD should consider another important aspect which is the representativeness ‘of collected samples’ with regard to ‘appropriate levels of concentrations in relation to EQS’ [7].

**Fig. 2:**
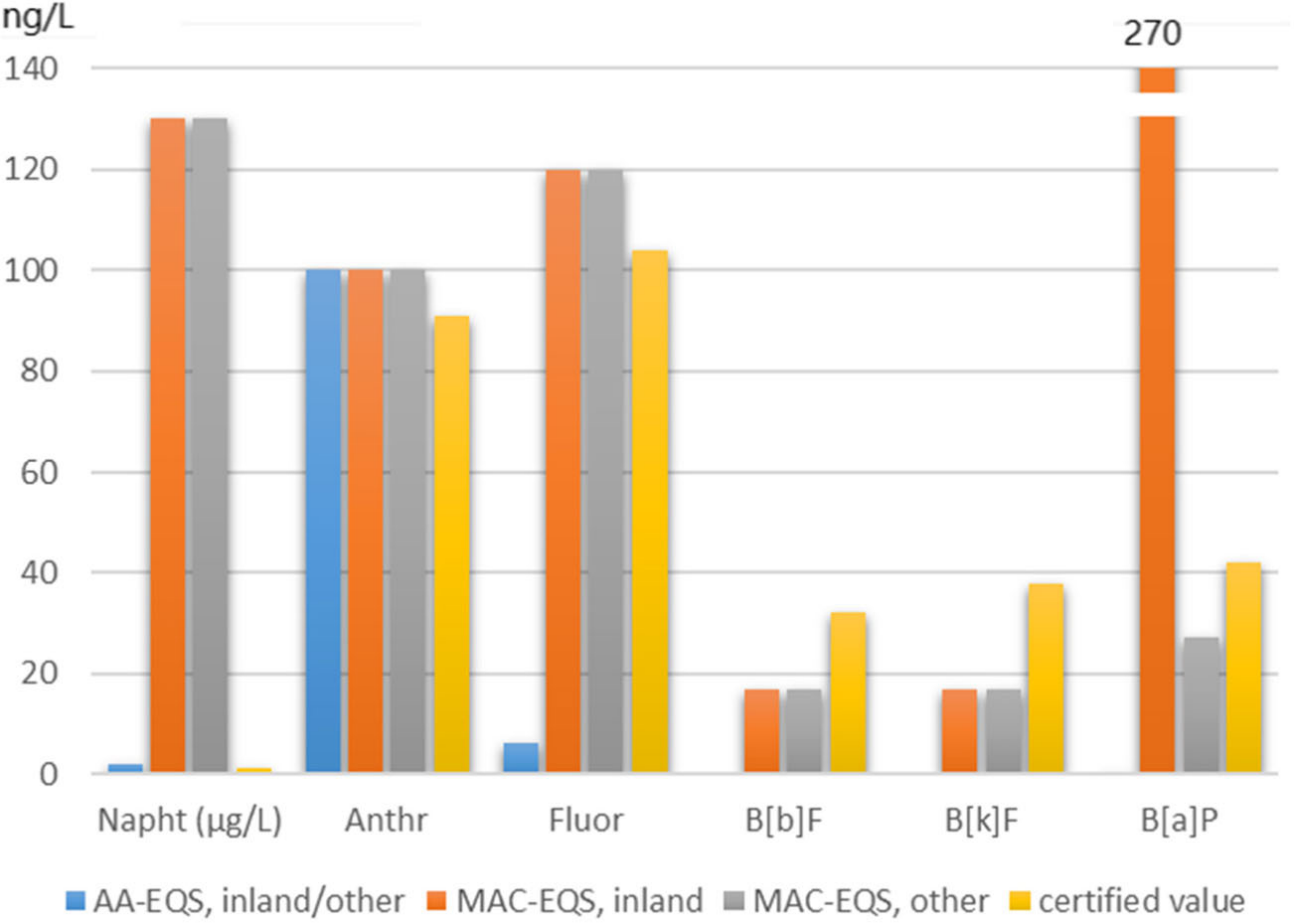
Comparison of ERM-CA100 certified values to Annual Average EQS (AA-EQS) and Maximum Allowable Concentration EQS (MAC-EQS) (Napht, naphthalene; Anthr, anthracene; Fluor, fluoranthene; B[b]F, benzo[*b*]fluoranthene; B[k]F, benzo[*k*]fluoranthene; B[a]P, benzo[*a*]pyrene) [7]

In the case of ERM-CA100, it is evident from a comparison of the certified values to the established EQS values (Table 2 and Fig.2) that several PAH contents match both the Annual Average EQS (AA-EQS) and the Maximum Allowable Concentration EQS (MAC-EQS) sufficiently well (i.e. for anthracene, benzo[*b*]fluoranthene and benzo[*k*]fluoranthene). No AA-EQS have been set for benzo[*b*]fluoranthene and benzo[*k*]fluoranthene, because benzo[*a*]pyrene is considered as marker for them since 2013. Others show differences, either with the MAC-EQS (i.e. for naphthalene) or with the AA-EQS (for fluoranthene). The content of benzo[*a*]pyrene is comparing well with the MAC-EQS for other surface waters (as defined in [5]) and to a lower degree with this parameter for inland waters, while it is much higher than the AA-EQS (0.17 ng/L).

The design of ERM-CA100 had been planned before the revision of the EQS values was published [5]. This is one of the reasons for the difference in matching certain EQS values. The match was anyway difficult to achieve for some PAHs, given the performance limitations of the analytical methods. This is equally true for several other organic PS, for which the water EQS are extremely low (e.g. PBDEs, bifenox, cypermethrin, dichlorvos, dicofol, PFOS). Currently available analytical methods are hardly capable of providing an accurate quantification at these concentration levels [42].

A very valuable feature of the characterisation study for ERM-CA100 is the combination of a variety of analytical measurement principles applied by the participants in this exercise (Table S2 in ESM). They used a sample pre-treatment based on two different principles [liquid-liquid extraction (LLE) and SPE] as well as different separation and quantification methodologies (HPLC-FLD and GC-MS). This contributes to demonstrate the absence of systematic measurement bias of the certified values and increases their usefulness. The suitability of this particular CRM with respect to the ‘representativeness of collected samples’ [7] is supported by the fact that the analytical methods used in the characterisation study are routinely applied for measuring PAHs in water samples, confirming that ERM-CA100 behaves analytically like a real environmental sample.

The relative expanded uncertainties for the certified values of ERM-CA100 are in the range of 10 to 25% and may be considered as large when compared to what is usually achieved when producing CRMs. However, they were considered acceptable for such a complex CRM matrix. The four PAHs with expanded uncertainties on the concentration values between 18 and 25% are the ones certified at very low concentrations, close to the limit of quantification for some of the laboratories participating in the characterisation. This might be the main factor behind such larger uncertainties.

A preliminary uncertainty estimation of the ready-to-use whole water RMs for PAHs prepared within the ENV08 project provided values in a similar range (9–21%, even without the contribution of long-term stability) [28], showing that the between-bottle heterogeneity makes up a significant contribution for this kind of materials. Similar uncertainty levels were also estimated for analytical measurement results on PBDEs and TBT in water RMs.

In conclusion, whatever approach is chosen, it is difficult to keep low the uncertainties of assigned values to the content of organic priority substances in a whole water (C)RM.

## Lessons learned in whole water analysis: the case of ERM-CA100 characterisation

To obtain one sample of the CRM, an easy-to-apply reconstitution protocol (enclosed in the certificate of the CRM [33], for details see ESM) had to be followed by the laboratories participating to the characterisation exercise. The data evaluation for the CRM value assignment provided some very interesting insights on the analysis of PAHs in non–filtered water, confirming observations already reported in the literature about the adsorption behaviour of PAHs and its consequences [15, 30].

**Fig. 3:**
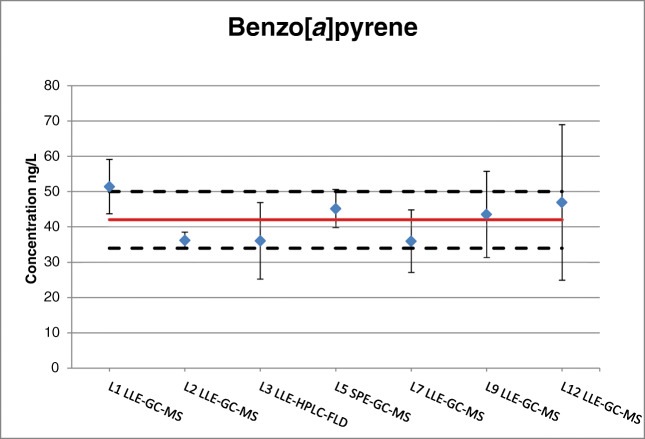
Certified value (solid line) and expanded uncertainty (dashed lines) of benzo[*a*]pyrene in ERM-CA100; the blue diamonds correspond to the laboratories’ means; the bars represent the expanded measurement uncertainty as reported by each laboratory

The use of an internal standard (i.s.) proved to be indispensable to compensate for the loss of PAHs adsorbed onto the humic acids [1]. One laboratory, which did not add any i.s., failed in measuring the PAHs accurately and its dataset was excluded from the assignment of the certified values (laboratory L4, Table S2 in ESM). All other laboratories used labelled PAHs as i.s., with the exception of laboratory L3 which employed B-B binaphthyl (using LLE coupled with HPLC-FLD). Applying a non-labelled i.s. did not necessarily diminish the accuracy of the measurement result per se. A closer look at the benzo[*a*]pyrene determination in Fig. 3 reveals no significant negative influence on data accuracy for laboratory L3, which employed a non-labelled internal standard. This laboratory reported uncertainties that were generally larger than the average, yet comparable to the other laboratories employing an analytical method also comprising of LLE extraction and HPLC-FLD but using a labelled i.s. instead.

**Fig. 4:**
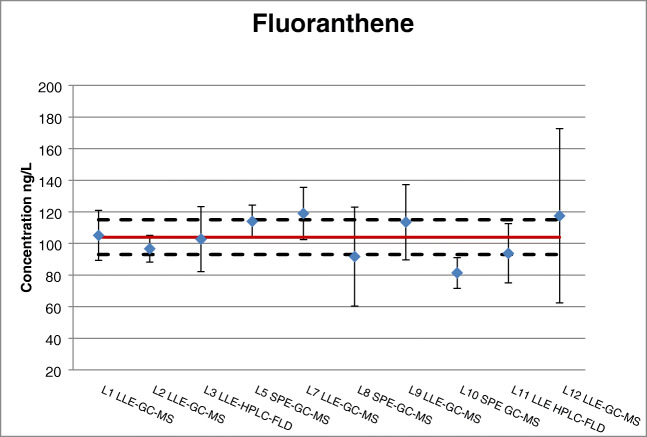
Certified value (solid line) and expanded uncertainty (dashed lines) of fluoranthene in ERM-CA100; the blue diamonds correspond to the laboratories’ means; the bars represent the expanded measurement uncertainty as reported by each laborator

Two extraction methodologies had been employed: two-thirds of the laboratories used LLE and the rest applied SPE (for the details refer to Table S2 in ESM). One of the performance criteria for the acceptance of technically valid characterisation data was a maximum bias of 30% related to the theoretical values of the concentration of the PAHs in the reconstituted water sample (as obtained from calculating the concentration in water upon spiking). Most of the non-compliant cases regarding this criterion originated from laboratories using SPE as extraction method. This is partially in line with the report from Wolska [35], where SPE was mentioned as potentially similar to LLE with regard to recovery, except for possible issues occurring during the transfer of the sample to the SPE column. Among the three laboratories using SPE and reporting technically valid datasets (see Fig. 4 with fluoranthene as example), two of them used SPE disks (L8 and L10), which seem to be one of the best choices for the extraction of organic substances from ‘whole water’ samples containing SPM [27, 32]. All details about the analytical methods employed by the laboratories participating to the characterisation are reported in Table S2 (see ESM).

The performance of the laboratories participating to the characterisation exercise is reflecting the state-of-the-art in determining PAHs at low concentration in whole water samples and it seems to indicate that there is room for improvement. Looking at the RSD of the characterisation datasets (which is the basis of the uncertainty component of the certified value related to characterisation u_char_, ESM Table S1), the range varies between 14 and 31% for the PAHs present at ng/L concentration. The only exception is benzo[*a*]pyrene with a RSD of 10% for its measurement data. This is an encouraging outcome, as benzo[*a*]pyrene remains in the 2013 Directive as the only PAH to be monitored as indicator for the group including benzo[*b*]fluoranthene, benzo[*k*]fluoranthene, indeno[1,2,3-*cd*]pyrene and benzo[*ghi*]perylene.

When comparing the certified values with the theoretical values for the reconstituted sample calculated upon spiking with the PAHs-containing solution B, most of them agree within the uncertainty interval. A difference is flagged for the content of benzo[*a*]pyrene and indeno[1,2,3-*cd*]pyrene (considering 2–3% as appropriate relative expanded uncertainty for the PAH concentrations in the original spiking solution B, which was prepared by substitution weighing and volumetric dilution). Their certified values are significantly lower than the theoretical ones. Such a non-complete recovery of the heavier PAHs from a sample had been reported earlier [1, 15, 16] and is attributed to their stronger adsorption onto humic acids compared to lighter PAHs. The same finding was observed in the IMEP-23 interlaboratory comparison (PAHs in water in the presence of humic acids) [30]. One of the laboratories that was expected to contribute to the reference values for the ILC reported values in disagreement with the gravimetric values for the three larger congeners, namely benzo[*a*]pyrene, indeno[1,2,3-*cd*]pyrene and benzo[*ghi*]perylene. This was explained by the absence of a sufficient equilibration after spiking with the labelled i.s., which caused an underestimation of the native congener. To our knowledge, all laboratories followed the reconstitution protocol in the characterisation study of ERM-CA100, which prescribed a 24-h equilibration time before analysis. This time was set based on previous studies [15] to allow for a complete equilibration of the sample with regard to the adsorption of the i.s. as well as the added PAHs spike onto the humic acids, which would lead to a full recovery of the analytes. However, this time was apparently still not sufficient for benzo[*a*]pyrene and indeno[1,2,3-*cd*]pyrene. It should be noted that the load of organic carbon in the IMEP-23 samples was 5 mg/L, i.e. much lower compared to the 20 mg/L in the ERM-CA100 material.

These observations confirm that PAHs (and all organic compounds that share a similar hydrophobicity) adsorb strongly to organic matter. Consequently, an analysis of the dissolved phase only does not reflect the actual load of apolar PS in the water body. Methods of analysis that can deal with ‘whole water’ samples are therefore imperative to use in the monitoring of organic PS in surface waters.

## Why is ‘whole water’ analysis not yet the general practice?

Literature records indicate that the four CEN standard methods for PAHs, PBDEs, TBT and OCP have not been widely employed so far by laboratories for monitoring organic pollutants in surface waters. This perceived reluctance to apply analytical methods for ‘whole water’ analysis was confirmed in a workshop organised by the JRC (1–2 March, 2018) for Member State experts. The aim of the workshop was to exchange experiences and knowledge on the analytical methods to be used for the analysis of the WFD Watch List (WL) substances [43]. An intense discussion evolved on the WFD ‘whole’ water requirements. Despite the mandatory analysis of such samples, many Member States professed the use of analytical methods embedding a filtration of water samples (e.g. for direct injection into LC-MS/MS). Such an approach could be acceptable for analysing *polar* organic substances (i.e. some of the WL substances), however not for more lipophilic compounds like PBDEs, PAHs, 17α-ethinylestradiol and 17β-estradiol or even for medium-polar compounds like nonylphenol [44, 45].

So far, there is a lack of reliable information on such pollutants to allow an extrapolation on the basis of the concentration in the dissolved fraction to the concentration in ‘whole water’ and more investigations should be carried out on the partitioning mechanisms and coefficients [14, 18]. For the time being, the analysis of the WFD organic priority substances has to be performed on ‘whole water’.

The assignment of biota EQS to some PS [5], including fluoranthene and benzo[*a*]pyrene, acknowledges the fact that hydrophobic substances may be difficult to determine in water. On the other hand, the Directive still allows flexibility for Members States to opt for water as matrix should this be advantageous for their monitoring strategy. Therefore, CRMs such as ERM-CA100 are important.

## Conclusions and outlook

The end of the road to the ‘ideal’ whole water CRM for organic priority substances is still some miles away. The ultimate goal is to accomplish the production of a water sample including both suspended particulate matter and humic acids as part of the water matrix. Pioneering this path, ERM-CA100 was the first CRM produced for the analysis of PAHs in water (and the only available so far) and a milestone in achieving at least a partial realisation of a ‘whole water’ sample, as requested by the WFD. The certified values match sufficiently well the WFD EQS for most of the congeners. Reference materials such as ERM-CA100 should be applied as QA/QC tools by the monitoring laboratories in their method validation and in their performance assessment of analytical methods for the analysis of PAHs in ‘whole water’ samples. The future design of whole water CRMs should consider the possibility of targeting also effect-based assays. The use of effect-based methods in the monitoring of surface water has been increasing in the last years mostly as screening tools, given that their calibration does not achieve the same level of reliability than more traditional GC- and LC-MS based methods [46–49].

There has been so far a limited demand for ERM-CA100 since its release in 2016. While an increased promotion could help, the main reason for the limited demand seems to be a reluctance of the measurement community to challenge their routine ways of analysis (notwithstanding the legislative requirements) with more realistic validation samples.

The JRC is currently working on producing ‘whole water’ CRMs containing suspended particulate matter by following the methodology developed during the ENV08 project [28].

At the same time, the monitoring community should acknowledge the necessity of such endeavours and increase its efforts in advancing the analytical state-of-the-art towards the development and validation of methods capable of managing such complex samples. The availability of ‘whole water’ CRMs would be of tremendous help for this purpose and would support the correct implementation of the water monitoring requirements of the WFD.

## Supplementary Information

ESM 1 (DOCX 24 kb)
