# Supplementary material for: A journey towards whole water certified reference materials for organic substances: measuring polycyclic aromatic hydrocarbons as required by the European Union Water Framework Directive
Source: Anal Bioanal Chem. 2021 Feb 18;413(9):2283–93. doi: 10.1007/s00216-021-03200-2 (PMC7987604; doi:10.1007/s00216-021-03200-2)
Supplement: Supplementary file 1 — (DOCX 24 kb) [file 216_2021_3200_MOESM1_ESM.docx]

**Supplementary Information**

A journey towards whole water certified reference materials for organic substances: measuring Polycyclic Aromatic Hydrocarbons as required by the European Union Water Framework Directive

*Ioannis Dosis*^1^, Marina Ricci**^🖃^*, Håkan Emteborg, Hendrik Emons*

European Commission, Joint Research Centre (JRC), 2440 Geel, Belgium

^1^Present address: German Environment Agency, Wörlitzer Platz 1, 06844 Dessau-Roßlau, Germany

*: These authors contributed equally to this work

^🖃^Corresponding author:

Marina Ricci

European Commission, Joint Research Centre (JRC), Geel, Belgium

Tel.: +32-14-571-962

Fax: +32-14-571-548

E-mail address: [marina.ricci@ec.europa.eu](mailto:marina.ricci@ec.europa.eu)

**ERM-CA100 Reconstitution Protocol**

The ERM-CA100 set contains:

A bottle with at least 1 L water

An ampoule with at least 24 mL HA spiking solution A (water)

An ampoule with at least 2 mL PAH spiking solution B (acetonitrile)

Recommended glassware (use of glassware should take place in a temperature range that complies with their calibrated volume):

- Volumetric glass flask, volume 500 mL, with glass stopper

- Volumetric glass pipette Class A, volume 1 mL

- Volumetric glass pipette Class A, volume 10 mL

- Volumetric glass pipette Class A, volume 2 mL

Reconstitution protocol:

1. Transfer approximately 450 mL of the water into the 500 mL volumetric glass flask.

2. Shake spiking solution A (humic acids solution) manually for approximately 1 minute.

3. Open the ampoule of spiking solution A and pipette 12 mL into the volumetric glass flask.

4. Shake the volumetric flask manually and gently (also by inversion) for approximately 1 minute.

5. Pipette 1 mL from the spiking solution B (PAH solution) into the volumetric glass flask.

N.B.: the pipette tip ought to be immersed below the water surface (to avoid loss of the most volatile PAHs) inside the volumetric glass flask until solution is delivered (flow out from the pipette by gravity) and slowly pulled back.

6. Mix gently by inversion of the volumetric glass flask for 1 minute.

7. Fill the volumetric glass flask with water up to the calibration mark.

8. Mix gently by inversion of the volumetric glass flask once again for 1 minute to achieve good homogeneity.

9. Leave the solution for about 24 hours in a dark place at + 4 °C ± 3 °C.

10. After this period, mix gently by inversion of the volumetric glass flask once again for 1 minute and proceed with the analysis according to the laboratory procedure within 24 hours.

N.B.: During the operations of mixing of the volumetric glass flask (4, 6, 8, and 10) pay attention not to lose drops of sample (e.g., due to leaking stoppers).

Table S1: Relative uncertainty contributions from characterisation (*u*_char_), homogeneity (*u*_bb_), short term and long term stability (*u*_sts_ and *u*_lts_) and final expanded uncertainty for the certified values of ERM-CA100

| Analyte | *u*_char, rel_ [%] | *u*_bb, rel_  [%] | *u*_sts, rel_  [%] | *u*_lts, rel_  [%] | *U*_CRM, rel_ [%] |
| --- | --- | --- | --- | --- | --- |
| Naphthalene | 4.1 | 2.8 | 1.0 | 1.4 | 10.3 |
| Anthracene | 4.7 | 2.7 | 0.8 | 1.8 | 11.4 |
| Fluoranthene | 3.8 | 1.7 | 0.5 | 2.6 | 9.9 |
| Benzo[*b*]fluoranthene | 8.8 | 4.7 | 2.0 | 8.0 | 25.5 |
| Benzo[*k*]fluoranthene | 6.0 | 1.9 | 0.8 | 8.6 | 21.3 |
| Benzo[*a*]pyrene | 5.6 | 4.2 | 1.3 | 5.4 | 17.6 |
| Indeno[1,2,3-*cd*]pyrene | 6.4 | 3.3 | 1.3 | 8.2 | 22.0 |

^1)^ Expanded (*k* = 2) uncertainty

**Table S2:** Summary of methods used in the characterisation study

| **Laboratory code – method** | **Sample pre-treatment** | **Analytical method** | **Type of calibration**  **Calibrants’ details** | **LOQ [ng/L]** |
| --- | --- | --- | --- | --- |
| **L1-GC-MS** | LLE* (hexane), extract concentration | GC-MS  Labelled PAHs added as internal standard | External: 1000/ 600 / 300 / 100 / 60 / 30 / 10 ng/mL PAH standard mix  PAH standard mix, Promochem, DE-Prom16, 100 µg/mL | 10 |
| **L2-GC-MS** | LLE (dichloromethane), extract clean-up with silica/alumina column | GC-MS  Labelled PAHs added as internal standard | External: 1-point calibration - concentration ca.1 µg/mL  NIST 2260A | 10 |
| **L3-HPLC-FLD** | LLE (*n*-pentane, 2-propanol), extract concentration with silica column | HPLC-FLD  B-B binaphtyl used as internal standard | External: 0.031 - 480 ng/mL  NIST SRM 1649 e, 0.79-19.85 mg/L | 0.1–5 |
| **L5-GC-MS** | SPE* (PLRP-S 15 - 25 Um) and clean-up | GC-MS  Labelled PAHs added as internal standard | Internal: 0.5 μg/L in water  Accustandard PAH mix 2 mg/mL | 10-20 |
| **L6-GC-MS** | LLE (hexane, acetone, dichloromethane), extract concentration | GC-MS  Labelled PAHs added as internal standard | External: 1, 2, 5, 10, 25, 50, 100, 250, 500, 1000 ng/mL  Dr. Ehrenstorfer Mix 9 100 µg/mL cyclohexane, CIL 100 µg/mL nonane | 10 |
| **L7-GC-MS** | LLE (cyclohexane), extract concentration | GC-MS  Labelled PAHs added as internal standard | Internal: Naphthalene 500 / 550 / 600 / 650 / 700 ng/mL each; ^13^C-Naphthalene 600 ng/mL  7 PAHs 5 / 20 / 35 / 50 / 65 ng/mL; ^13^C-PAHs 35 ng/mL  Dr Ehrenstorfer, 16 PAHs in cyclohexane, 10 ng/µL each CIL, 100 µg/mL in *n*-nonane | 1-10 |
| **L8-GC-MS** | SPE (SPEDEX, automatic SPE with DVB disc) and clean-up | GC-MS  Labelled PAHs added as internal standard | Internal: 5-10-25-50-100-250-500 ng/mL  PAH-mix 45, Dr Ehrenstorfer 10 ng/µL in ACN | 1 -40 |
| **L9-GC-MS** | LLE (acetone / petroleum ether 40-60), extract concentration | GC-MS  Labelled PAHs added as internal standard | External: 3 points calibration curve  Accustandard S-21969-R1-100X-5mL | 0.07-30 |
| **L10-GC-MS** | SPE (Speedisk Extraction Disk H_2_O-philic DVB) and clean-up | GC-MS  Labelled PAHs added as internal standard | External: (repeated one point calibration): 8-200 ng/mL calibration Range: 0-1000 ng/mL extract  16 EPA PAH: NIST SRM1649e 8-200 ng/mL | 0.2–4.4 |
| **L11-HPLC-FLD** | LLE (hexane), extract clean-up with silica/alumina column | HPLC-FLD  Labelled PAHs added as internal standard | External: 9-point calibration - concentration ca.1 - 500 ng/mL  Dr. Ehrenstorfer,(ref. L20950009AL) (CAS:75-05-8) 10 μg/mL | 2 |
| **L12-GC-MS** | LLE (toluene), extract clean-up with silica/alumina column | GC-MS  Labelled PAHs added as internal standard | External: 0.0032, 0.016, 0.08, 0.8, 4, 10 µg/mL  PAH-Mix, Dr. Ehrenstorfer, original 10 µg/mL, diluted to 1 µg/mL | 0.1–3.7 |
| **Not used for value assignment** | | | | |
| **L4-LC-APPI-MS/MS** | SPE [ENVI-18 (by Supelco)] and clean-up | LC-FL quantification  LC-APPI-MS/MS confirmation/identification  No internal standard | External: 1, 5, 10, 25, 50, 75 & 100 ng/mL except for naphthalene and fluoranthene for which are 10, 50, 100, 250, 500, 750 & 1000 ng/mL  A standard solution (S1), mixture of 8 PAHs is prepared at a concentration of 1 μg/mL by mixing appropriate volumes of individual standard solutions of each PAH with concentration of 100 μg/mL | 10–100 |

* LLE: Liquid-liquid extraction, SPE: Solid phase extraction
